# Colonic Metastasis of Adenoid Cystic Carcinoma 19 Years after the Primary Tumor Resection

**DOI:** 10.1155/2021/5511935

**Published:** 2021-04-27

**Authors:** Subramanya Sakaleshpura Mallikarjunappa, Shriram Jakate, Lin Cheng

**Affiliations:** Department of Pathology, Rush University Medical Center, Chicago, IL 60612, USA

## Abstract

Adenoid cystic carcinoma (ACC) is a tumor characterized by slow growth and late distant metastasis. The lung and breast are the most common sites for metastasis. Colonic metastasis of such a tumor is rare, with few case reports available. Here, we report a case of ACC arising from minor salivary gland that metastasized to the colon 19 years after the primary tumor resection, with literature review of the clinical, histological, and molecular features of ACC. This case raises our awareness of such tumors as a differential diagnosis of colorectal cancer.

## 1. Introduction

Metastatic tumors involving colon are rare, accounting for only 0.338% of all colorectal malignancies in a large multi-institutional study, when lymphoproliferative diseases, direct invasion, and mesenteric and peritoneal spreading are excluded [1]. The most common primary site of colorectal metastasis is the breast, followed by the lung and malignant melanoma [1]. Metastatic adenoid cystic carcinoma (ACC) in the colon is rare, with only a few case reports available [2–4]. Here, we report a case of ACC metastatic to the colon 19 years after resection of the primary tumor, to broaden the differential diagnosis of colorectal malignancies and to better understand the clinical, histological, and molecular features of ACC.

## 2. Case Presentation

This is a 78-year-old man who was diagnosed with ACC in oral cavity 19 years ago and underwent resection. The primary tumor was 4 cm in size, with multiple positive margins and lingual nerve involvement. The lymph nodes were all negative. He received adjuvant radiation. He was tumor free until 9 years ago when multiple small nodules in the right lung were identified. Biopsy revealed metastatic ACC, and all the nodules were enucleated. The patient developed a new left lung mass 5 years ago, which was diagnosed as a primary lung adenocarcinoma.

**Figure 1:**
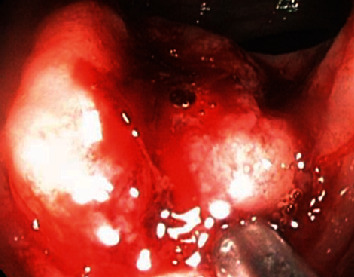
Colonoscopy showing a 4 cm firm mass firm with a depressed central aspect.

The patient presented for tumor surveillance and underwent a whole body PET CT scan. A solitary region of increased metabolic activity was identified in the ascending colon, with the maximum SUV 3.6. A colonoscopy was performed, and a mass was found in the ascending colon (Figure 1). Saline injection did not raise the mass. Biopsy was performed and revealed metastatic ACC. The overlying mucosa showed no dysplasia.

The patient then received a right hemicolectomy. The tumor was located in the ascending colon, 3 cm to the ileocecal valve, 2.3 cm in size, firm, red-tan, and with central umbilication (Figure 1). The resection specimen also contained an unremarkable appendix and eighteen negative lymph nodes.

**Figure 2:**
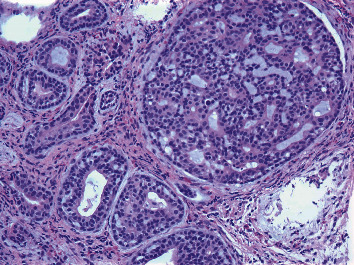
Microphotograph (200x) of the mass showing a very distinctive appearance and consists of different patterns. There were smaller gland or duct-like structures as well as larger nests with cribriform pattern. Some glands or spaces showed mucin-like or basophilic material. The tumor cells were round to oval with fine chromatin, small inconspicuous nucleoli, indistinct cell borders, and light nongranular cytoplasm. Mitotic figures were rare.

Microscopically, the tumor showed cribriform and tubular structures, with lightly basophilic material in the lumen. Some tubes had double layers, with inner layer of epithelial cells and outer layer of myoepithelial cells. At higher magnification, the tumor cells showed minimal cytological atypia, scant cytoplasm, fine chromatin, small inconspicuous nucleoli, and rare mitotic figures (Figure 2).

**Figure 3:**
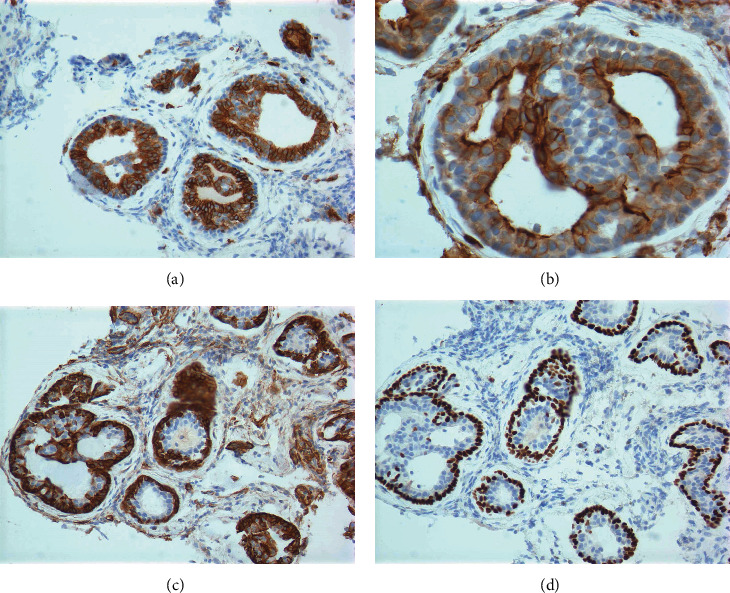
(a) C-kit (100x), (b) CEA (400x), (c) SMA (200x), and (d) P63 (200x), respectively. The luminal epithelial cells of the tumor are positive for C-kit and CEA; the abluminal myoepithelial cells of the tumor are positive for SMA and P63.

Immunohistochemically, the tumor demonstrated epithelial-myoepithelial dual lineage differentiation, which confirmed the diagnosis of metastatic ACC. The luminal epithelial cells were positive for cytokeratin AE1/AE3, C-kit, and CEA, while the abluminal myoepithelial cells were positive for P63 and SMA (Figure 3). The tumor cells were also negative for Synaptophysin, Chromogranin, TTF-1, CK20, and CDX2, excluding carcinoid tumor, metastatic lung adenocarcinoma, and primary colorectal adenocarcinoma. The Ki-67 immunostain showed a proliferation index of 10%.

## 3. Discussion

ACC is considered as a salivary gland tumor. The most common site is within the oral cavity [5]. Primary ACC can also present in upper aerodigestive tract, lung, breast, and cervix, but never in colon.

ACC can occur between 10 and 96 years of age with peak in the 6th decade of life [6, 7]. Patients usually experience gradually increased local swellings and tenderness. A common treatment strategy is surgical resection followed by adjuvant radiation, and disease-specific survival (DSS) at 5 years can reach 89% [8]. However, ACC is famous for its late distant metastasis and poor long-term prognosis. DSS at 15 years was only 40% [9]. When ACC is identified in the colon, efforts should be made to look for distant history of primary ACCs, such as 19 years ago in our patient. Interestingly, in one of the publications, the patient only had remote history of mandibular “abscess,” which could be an undiagnosed ACC [4].

The pathway of ACC metastasis is mainly hematogenous. The most commonly involved organ is the lung, followed by the liver and bone. It is not clear why metastasis to the colon is so rare. Occasionally, the tumor can directly extend to local lymph nodes, but spreading through lymphatics to regional lymph nodes is extremely rare. When a large nerve is involved, tumor cells can also spread along the nerve sheath. Multiple factors can predispose to the metastasis in ACC, such as large tumor size, positive margins, and large nerve involvement [10]. Our patient had multiple positive margins and lingual nerve involvement, which could contribute to his multiple metastases.

ACC has a unique histology. As shown in our case, the tumor is composed of epithelial and myoepithelial cells. Both cell types are cytologically bland, basaloid, with hyperchromatic nuclei, and scant pale to clear cytoplasm. Three growth patterns can be seen: cribriform, tubular, and solid. Cribriform pattern is the classic one, with a Swiss cheese-like appearance at low magnification. The punched-out spaces are mainly pseudolumen filled with hyalinized basement membrane-like material and/or basophilic amorphous glycosaminoglycans. However, true lumen can coexist. Solid growth pattern correlates with higher tumor grading and worse prognosis.

Metastatic ACC in the colon should be differentiated from primary colonic adenocarcinoma with the aid of immunohistochemical studies. ACC shows dual lineage differentiation: the epithelial cells are positive for C-kit, EMA, and CEA; while the myoepithelial cells are positive for P63, SMA, Calponin, S100, Vimentin, and GFAP [11]. The epithelial and myoepithelial cells of ACC also share some common immunophenotypes, such as positive for pancytokeratin AE1/AE3, CK7, CK14, CK17, CK19, P53, and Bcl-2 [12]. On the contrary, primary colonic adenocarcinoma usually shows single lineage differentiation and is diffusely positive for CK20 and CDX2 and negative for CK7.

ACCs show genetic heterogeneity. The most common genetic alteration in ACC is a MYB-NFIB translocation [13], which can be found in 86% of adenoid cystic carcinomas. Another common genetic alteration is PI3K. MYB fusions are associated with higher 10-year overall survival rate while PI3K alterations are associated with a longer disease-free interval [14]. Comparing with the primary tumors, metastatic ACCs show enriched genetic mutations and higher intratumoral genetic heterogeneity [15].

Since target therapies or immunotherapies for ACCs are still under investigation, the current treatment options of metastatic ACC include local control by surgery or radiation and systemic management with chemotherapy.
